# Outcomes and Utilization of Therapeutic Hypothermia in Post-Cardiac Arrest Patients in Teaching Versus Non-Teaching Hospitals: Retrospective Study of the Nationwide Inpatient Sample Database (2016)

**DOI:** 10.7759/cureus.9545

**Published:** 2020-08-04

**Authors:** Anil Jha, Ajit Thota, Kevin G Buda, Akshay Goel, Ashish Sharma, Anand M Krishnan, Hardik K Patel, Fangcheng Wu

**Affiliations:** 1 Internal Medicine, Lawrence General Hospital, Lawrence, USA; 2 Internal Medicine, Carney Hospital, Dorchester, USA; 3 Internal Medicine, Hennepin County Medical Center, Minneapolis, USA; 4 Internal Medicine, University of Arkansas for Medical Sciences, Little Rock, USA; 5 Internal Medicine, Yuma Regional Medical Center, Yuma, USA; 6 Internal Medicine, University of Connecticut Health, Farmington, USA; 7 Internal Medicine, Mount Sinai Hospital, New York, USA; 8 Internal Medicine, Memorial Healthcare, Hollywood, USA

**Keywords:** cardiac arrest, therapeutic hypothermia, mortality, length of stay, hospitalization charges, nis

## Abstract

Background

Using therapeutic hypothermia (TH) reduces the core body temperature of survivors of cardiac arrest to minimize the neurological damage caused by severe hypoxia. The TH protocol is initiated following return of spontaneous circulation (ROSC) in non-responsive patients. Clinical trials examining this technique have shown significant improvement in neurological function among survivors of cardiac arrests. Though there is strong evidence to support TH use to improve the neurologic outcomes in shockable and nonshockable rhythms, predictors of TH utilization are not well-characterized. Our study tried to evaluate TH utilization, as well as the effect of the teaching status of hospitals, on outcomes, including mortality, length of stay, and total hospitalization charges.

Method

We conducted a retrospective analysis of the Healthcare Cost and Utilization Project - Nationwide Inpatient Sample (HCUP-NIS) database. Patients with an admitting diagnosis of cardiac arrest, as identified by the corresponding International Classification of Disease, 10^th^ Revision (ICD-10) code for the year 2016 were analyzed. In addition, we identified TH using the ICD-10 procedure code. A weighted descriptive analysis was performed to generate national estimates. Groups of patients admitted to teaching hospitals were compared to those admitted in non-teaching hospitals. Patients were stratified by age, sex, race, and demographic and clinical data, including the Charlson Comorbidity Index (CCI), for these two groups, and statistical analysis was done for the primary outcome, in-hospital mortality, as well as the secondary outcomes, including length of stay (LOS) and total hospitalization charges. Fisher’s exact test was used to compare proportions and student’s t-test for continuous variables. Statistical analysis was completed by linear regression analysis.

Results

A total of 13,780 patients met the inclusion criteria for cardiac arrest admission. The number of patients with cardiac arrest admitted to a teaching hospital was 9285. A total of 670 patients received TH, with 495 admissions to teaching hospitals. The population of females in the hypothermia group was 270. The mean age of patients received TH was 59.4 years. In patients who received TH, 65% were Caucasians followed by Hispanics (16%), with no significant statistical racial differences in groups (p=0.30). The majority of patients with TH in both groups (teaching vs. non-teaching admissions) had Medicare (58.8% vs 49.5%; p=0.75). Hospitals in the southern region had the most admissions in both groups (45.7% and 31.3%), with the northeast region having the least non-teaching hospital admissions (8.5%) and approximately similar teaching hospital admissions in other regions (~22%) (p=0.27). The total number of deaths in this group was 510, out of which 370 were in a teaching hospital. After adjusting for age, sex, race, income, the CCI, hospital location, and bed size, mortality was not significantly different between these two groups (p=0.797). We found increased LOS in patients admitted to teaching hospitals (p=0.021). With a p-value of 0.097, there were no differences in total hospitalization charges in both groups.

Conclusion

There were no significant differences in mortality or total hospitalization charge between patients admitted with cardiac arrest to a teaching hospital and received TH as compared to a non-teaching hospital although patients admitted to teaching hospitals stayed longer.

## Introduction

This study aimed to understand the utilization of TH in post-cardiac arrest patients admitted to teaching vs. non-teaching hospitals, as well as the outcomes, including mortality, length of stay, and total hospitalization charges.

The annual incidence of out-of-hospital cardiac arrests (OHCA) in the United States is reported to be more than 340,000. Mortality is estimated to be nearly 90% and approximately 9% of the survivors were noted to have good neurological function [1].

Therapeutic hypothermia (TH) has been shown to mitigate global cerebral ischemia and improve recovery from the neurological problems caused by severe hypoxia in cardiac arrest and neonatal asphyxia [2-4]. Since its initial description by Hippocrates in 400 BC (of its ability to reduce hemorrhage in the wounded), it was extended to be studied mainly as a neuroprotective measure in multiple settings such as after cardiac arrests, prolonged open-heart surgeries, traumatic brain injuries, and ischemic stroke [5].

After various animal models showed improved functional recovery and reduced cerebral histological defects with TH, this was also extended into human studies, with promising results [2-3,6-9]. The pathophysiologic basis for this benefit is believed to be multifactorial but mostly thought to be related to metabolic suppression and reduced cerebral oxygen demand with induced hypothermia [10-12].

With accumulating evidence from two major randomized and several non-randomized trials, the TH protocol was officially incorporated in post-resuscitation guidelines by the International Liaison Committee on Resuscitation (ILCOR) and the American Heart Association (AHA) in 2003 [2-3,13]. A strong level of evidence exists to recommend the use of TH in comatose, out-of-hospital cardiac arrest survivors whose initial rhythm was ventricular fibrillation or pulseless ventricular tachycardia. However, it is also being used regularly in survivors with an initial non-shockable rhythm (asystole/pulseless electrical activity) and in-hospital cardiac arrest situations based on the consensus of expert opinion.

After the initial introduction, the protocol did not receive widespread recognition, as the rate of implementation among physicians was reported to be only at 30%-40% [14]. Many barriers were recognized, and active measures and strategies were implemented to change this clinical practice. Though TH utilization remains variable, predictors of utilization are not well-understood.

Generally, teaching hospitals are big establishments, and they are considered as being well-equipped with resources and leading physicians among the staff. It is usually assumed that patients who are being treated in theses centers are getting high-quality health-care [15]. Therapeutic hypothermia utilization and its outcome is one of the surrogates of quality care provided by the hospitals. This review seeks to evaluate retrospectively the utilization rates and outcomes of the use of TH in teaching vs. non-teaching hospitals.

## Materials and methods

Study design and database description

This retrospective cohort study included patients hospitalized in 2016 due to a cardiac arrest. The Nationwide Inpatient Sample (NIS) database 2016 was used to select the patient population. We have chosen the 2016 dataset, as this was the only available current dataset with a fully integrated International Classification of Disease, 10th Revision, Clinical Modification code at the time of this study. The database is created and maintained by the Agency for Healthcare Research and Quality (AHRQ), which is one of the largest publicly available databases. It contains both patient and hospital-level information. There are 30 discharge diagnoses and 15 procedures recorded for each patient based on the International Classification of Disease, 10th Revision, Clinical Modification (ICD-10-CM). All non-federal acute care hospitals participate in this database. Hospitals are stratified according to bed size, teaching status, urban vs. rural location, as well as geographic region. As this is a stratified probability sample, a 20% sample of all hospitals within the stratum is collected. The discharges from the acute care hospitals are recorded and weighted to ensure they are nationally representative. In 2016, 4575 hospitals from 46 states plus the District of Columbia participated in the NIS database, covering more than 97% of the U.S. population [16].

Study patients

Patients admitted with a cardiac arrest were identified using ICD 10-CM codes. The ICD-10 codes were for cardiac arrests in the setting of cardiac conditions, with other specific and nonspecific conditions. As there is no definite code for shockable vs non-shockable rhythm, it was not included in our study. The ICD-10 procedure code was applied to identify the use of TH (Annexure 1). Patients with cardiac arrest who received TH were included in our study population.

Study variables

The NIS database identifies the participating hospitals according to their geographical location, rural vs. urban location, and teaching vs. non-teaching status, as well as per their size in terms of bed numbers. We included in our study the teaching and non-teaching status of hospitals for comparison.

This study also evaluated other confounders such as age, sex, race/ethnicity, the median yearly income for the patient’s zip code, patient’s comorbidities, hospital location (rural or urban), and geographic region (Northeast, Midwest, West, or South). For comorbidity, we used the Charlson Comorbidity Index (CCI) for administrative data and re-categorized it into groups as CCI=0, CCI=1, CCI=2, and CCI≥3. These variables were part of prior studies done on this topic as well.

Statistical analysis

The statistical analysis was performed using STATA version 15.1 (Stata Corp, College Station, Texas) to generate variance estimates and p-values. To calculate unadjusted odds ratios for primary and secondary outcomes, we used univariate linear regression analysis. We adjusted for the above-mentioned confounders using multivariate linear regression analysis. Fisher’s exact test was used to compare proportions and the student’s t-test for continuous variables. The p-value threshold for statistical significance was 0.05.

Outcomes

The primary outcome of this study was in-hospital mortality in patients admitted with out-of-hospital cardiac arrest and who received TH in teaching vs. non-teaching hospitals. We also reviewed the overall utilization of TH in these patients in the same setting. The in-hospital mortality is provided in the NIS database for each discharge. Further analysis was done to see the effect of the teaching status of hospitals on mortality as well as secondary outcomes, including the length of stay (LOS) and total hospitalization charges.

## Results

Patient characteristics and utilization of TH

There were 7,135,090 discharges in 2016 [15]. A total of 13,780 cardiac arrests were admitted in 2016. Six-hundred seventy patients received TH, with 495 admissions to the teaching hospital and a mean age of 59 years. The population of females in the TH group was 270. Of the patients who received TH, 65% were Caucasians followed by Hispanics (16%), with there being no significant statistical racial differences in groups (p=0.30). The majority of patients with TH in both groups (teaching vs non-teaching admissions) had Medicare (58.8% vs 49.5%; p=0.75). Hospitals in the southern regions had the most admissions in both groups (45.7% and 31.3%), with hospitals in the northeast having the least non-teaching hospital admissions (8.5%) and approximately similar teaching hospital admissions in other regions (~22%) (p=0.27). A majority of patients in teaching hospitals (34.3%), as well as non-teaching hospitals (45.7), had Charlson Comorbidity Index >3. Table 1 provides the detailed characteristics of our study population.

**Table 1:** Patient and hospital characteristics

	Total	Teaching Hospital	Nonteaching Hospital	p-value
Patient Characteristic:				
No.(%) of patients	670	495(73.8)	175 (28.2)	
Female, No. (%)		235(47.5)	35(20.0)	
Race/Ethnicity, No (%)				0.308
White		313 (63.3)	113 (64.5)	
African-American		82 (16.7)	17 (9.7)	
Hispanic		38 (7.8)	28 (16.1)	
Asian or Pacific Islander		32 (6.7)	6 (3.2)	
Native American		0 (0)	6 (3.2)	
Other		27 (5.6)	6 (3.2)	
Median age, Y, (95% CI)	63.9 (63.2-64.7)	59.7 (56.8-62.7)	58.5 (52.8-64.2)	
Charlson Comorbidity Index Score				0.483
0		94 (19.4)	35 (20.0)	
1		143 (29.2)	30 (17.1)	
2		84 (17.2)	30 (17.1)	
≥3		168 (34.3)	80 (45.7)	
Median annual income for patient's Zip code, US$, No. (%)				0.188
1-42999		148 (29.9)	31 (17.6)	
43,000 - 53,999		132 (26.8)	67 (38.2)	
54,000 - 70,999		107 (21.6)	57 (32.3)	
>71,000		107 (21.6)	16 (11.8)	
Insurance type, NO. (%)				0.751
Medicare		245 (49.5)	103 (58.8)	
Medicaid		88 (17.8)	31 (17.7)	
Private		145 (29.4)	36 (20.6)	
Uninsured		16 (3.16)	5 (2.9)	
Hospital characteristics				
Hospital region, No. (%)				0.272
Northeast		120 (24.2)	15 (8.6)	
Midwest		110 (22.2)	40 (22.9)	
South		155 (31.3)	80 (45.7)	
West		110 (22.2)	40 (22.9)

In-hospital mortality based on teaching status of hospital

Patients who were admitted to teaching hospitals with a cardiac arrest and received TH had a mortality rate of 75% (510 patients). Whereas overall mortality in cardiac arrest patients were 10,260 out of 13,780 admitted (74.5%).

Using a multivariate linear regression analysis that adjusted for patient and hospital-level confounders, we found the adjusted coefficient of mortality was -0.028, 95% CI -0.24-0.19, and p-value of 0.797. Patients without insurance (self pay) had decreased mortality (coefficient 0.43, p= 0.014, 95%CI 0.086-0.77). Patients admitted to a medium bed size hospital showed decreased mortality (coefficient -0.30, p=0.006, 95%CI -0.51 - -0.87). There was no effect of racial difference and hospital location or region on mortality. Detailed results are summarized in Table 2.

**Table 2:** Result of multivariate linear regression for primary outcome mortality Std err: Standard Error, Conf: Confidence

Mortality					
	Coefficient	Linearized Std. Err.	P	[95% Conf.	Interval]
Teaching hospital	-0.0282254	0.1097373	0.797	-0.2434768	0.1870261
Age	0.0003119	0.0031306	0.921	-0.0058288	0.0064525
Female	-0.0030135	0.0793216	0.97	-0.1586041	0.1525772
Race					
African American	0.0309733	0.1187131	0.794	-0.2018844	0.2638309
Hispanic	0.0580718	0.128843	0.652	-0.1946557	0.3107993
Asian or Pacific Islander	-0.1578534	0.1941726	0.416	-0.538726	0.2230192
Native American	0.1481538	0.1542157	0.337	-0.1543426	0.4506503
Other	0.0521277	0.147022	0.723	-0.2362582	0.3405135
Median annual income in patient’s zip code, US$ (reference 1 - 42,999)					
43,000 - 53,999	-0.0575723	0.1226186	0.639	-0.2980907	0.1829461
54,000 - 70,999	0.0584806	0.1168395	0.617	-0.1707019	0.287663
>71,000	0.1329478	0.1269793	0.295	-0.1161242	0.3820197
Primary Payer( reference medicare)					
Medicaid	-0.0341967	0.1259209	0.786	-0.2811924	0.2127991
Private	0.0810423	0.0855511	0.344	-0.0867675	0.248852
Self-pay	0.4293107	0.1749926	0.014	0.0860598	0.7725615
Charlson comorbidity score					
1	-0.0514546	0.1135377	0.65	-0.2741606	0.1712514
2	-0.026537	0.1335714	0.843	-0.2885394	0.2354653
≥3	0.0788199	0.0982482	0.423	-0.1138954	0.2715352
Urban hospitals (reference-rural)	-0.2374598	0.1429704	0.097	-0.5178985	0.0429789
Hospital region (reference Northeast)					
Midwest	0.1657511	0.0918772	0.071	-0.0144674	0.3459697
South	-0.0914026	0.1214781	0.452	-0.3296839	0.1468786
West	0.0708363	0.1075713	0.51	-0.1401664	0.281839
Hospital bed-size (reference small)					
Medium	-0.2974968	0.1075469	0.006	-0.5084518	-0.0865419
Large	-0.0990006	0.0932798	0.289	-0.2819704	0.0839693

The results related to secondary outcomes are as follows.

Hospital length of stay based on teaching status of the hospital

The median length of stay for patients who were admitted with a cardiac arrest and received TH in teaching hospitals was 4.33, whereas in non-teaching hospitals, it was 3.45. The multivariate linear analysis showed increased odds of length of stay in teaching hospitals as compared to non-teaching hospitals (coefficient=5.58, p=0.033, 95%CI 0.45-10.71). Patients with income group in the $43,000 - $53,999 range also showed increased length of stay (LOS) (coefficient=6.23, p=0.021, 95%CI 0.93-11.54). Detailed results are summarized in Table 3.

**Table 3:** Result of multivariate linear regression for length of stay Std err: Standard Error, Conf: Confidence; LOS: Length of Stay

LOS					
	Coefficient.	Linearized Std. Err.	P	[95% Conf.	Interval]
Teaching hospital	5.582218	2.614698	0.033	0.4534474	10.71099
Age	-0.0634208	0.0726223	0.383	-0.2058706	0.0790291
Female	2.766286	2.07937	0.184	-1.312431	6.845003
Race					
African American	0.0031588	1.631666	0.998	-3.197379	3.203697
Hispanic	8.418846	6.932309	0.225	-5.178987	22.01668
Asian or Pacific Islander	-2.016921	2.691396	0.454	-7.296138	3.262295
Native American	1.900837	3.823703	0.619	-5.599417	9.40109
Other	-1.705178	2.901342	0.557	-7.396207	3.985851
Median annual income in patient’s Zip code, US$ (reference 1 - 42,999)					
43,000 - 53,999	6.231577	2.704804	0.021	0.9260613	11.53709
54,000 - 70,999	1.931953	1.933166	0.318	-1.859982	5.723888
>71,000	1.742499	1.966798	0.376	-2.115406	5.600404
Primary Payer( reference Medicare)					
Medicaid	4.371632	3.769085	0.246	-3.021488	11.76475
Private	-0.7287483	2.159272	0.736	-4.964194	3.506698
Self-pay	-6.573342	3.493908	0.06	-13.4267	0.280014
Charlson comorbidity score					
1	2.153863	2.581188	0.404	-2.909178	7.216904
2	2.528446	2.493199	0.311	-2.362003	7.418895
≥3	5.91804	3.069004	0.054	-0.1018583	11.93794
Urban hospitals (reference-rural)	-2.211369	3.311612	0.504	-8.707148	4.284409
Hospital region (reference Northeast)					
Midwest	-2.047142	1.817454	0.26	-5.612107	1.517824
South	3.797854	2.049053	0.064	-0.2213945	7.817103
West	2.45962	3.572239	0.491	-4.547383	9.466623
Hospital bed-size (reference small)					
Medium	3.969381	2.613727	0.129	-1.157486	9.096247
Large	3.866044	1.984591	0.052	-0.0267629	7.758852

Total hospitalization charges based on the teaching status of the hospital

The median total hospitalization charges were $79,711 and $69,228 for patients admitted to teaching vs non-teaching hospitals, respectively. With the adjustment of other confounding variables, the total charge in both settings had no significant statistical difference (p=0.097). Patients with the income group in the $43,000 - $53,999 range also showed increased total hospitalization cost in teaching hospitals when compared to non-teaching hospitals (coefficient=117298, p=0.012, 95%CI 26266.84 - 208328.80). Detailed results are summarized in Table 4.

**Table 4:** Result of multivariate linear regression for total hospitalization charges Std err: Standard Error, Conf: Confidence

Total charges analysis					
	Coef.	Linearized Std. Err.	P	[95% Conf.	Interval]
Teaching hospital	76029.65	45598.81	0.096	-13415.38	165474.7
Age	-1170.897	1069.137	0.274	-3268.078	926.2844
Female	31963.85	31345.08	0.308	-29521.55	93449.26
Race					
African American	8567.286	26231.08	0.744	-42886.68	60021.25
Hispanic	170854.1	111429.1	0.125	-47721.25	389429.4
Asian or Pacific Islander	-51049.28	40473.35	0.207	-130440.4	28341.82
Native American	75253.86	63030.33	0.233	-48384.23	198892
Other	-33001.68	37694.12	0.381	-106941.1	40937.78
Median annual income in patient’s Zip code, US$ (reference 1 - 42,999)					
43,000 - 53,999	117297.8	46407.32	0.012	26266.84	208328.8
54,000 - 70,999	37797.24	32631.09	0.247	-26210.76	101805.2
>71,000	50941.5	34978.21	0.145	-17670.53	119553.5
Primary Payer (reference Medicare)					
Medicaid	68855.48	67385.52	0.307	-63325.58	201036.5
Private	-18418.36	33716.39	0.585	-84555.25	47718.53
Self-pay	-74584.7	50477.49	0.14	-173599.6	24430.16
Charlson comorbidity score					
1	28242.63	41859.66	0.5	-53867.8	110353.1
2	39060.21	38223.52	0.307	-35917.72	114038.1
≥3	69719.45	50398.13	0.167	-29139.75	168578.7
Urban hospitals (reference-rural)	-17705.55	57616	0.759	-130723.1	95311.98
Hospital region (reference Northeast)					
Midwest	-30406.24	29218.45	0.298	-87720.12	26907.64
South	28274.62	30272.88	0.35	-31107.61	87656.84
West	70030.03	60423.75	0.247	-48495.08	188555.1
Hospital bed-size (reference small)					
Medium	61012.17	44979.45	0.175	-27217.93	149242.3
Large	61100.67	35160.51	0.082	-7868.96	130070.3

## Discussion

There is limited evidence for the predictors of TH use in out-of-hospital cardiac arrest. The most recent study that was done with this database (2007-2010) and published in 2015 showed an increasing utilization of TH in general from 0.34% in 2007 to 2.49% in 2010 [17].

To the best of our knowledge, our study is the first to compare the outcomes of TH patients based on the teaching status of the hospitals. Several studies in the past have shown low utilization of TH [18-20].

Our study has found a significant increase in the overall utilization of TH to 4.86% (compared to the reported utilization in recent studies), out of which 3.60% were in teaching hospitals. Patients admitted to teaching hospitals were younger (mean age 59 years).

There is no study done to see the effect of the teaching status of hospitals on mortality. Although studies have shown better outcomes in general with the utilization of therapeutic hypothermia [21-23].

Our study has shown that teaching hospital status was not an independent predictor of in-hospital mortality.

There was no effect of race, median income, location, or regional variance on mortality in patients with cardiac arrest who received TH. This signifies that even with limited resources, non-teaching hospitals are doing well regarding the care of one of the sickest categories of patients.

The adjusted length of stay was marginally higher in the teaching status of the hospitals in patient groups that received TH (p=0.021). The literature search has not shown any study commenting on length of stay in patients who received therapeutic hypothermia. Although patients with cardiac arrest seem to stay longer given their level of sickness. This might require further study to identify the underlying causes.

An important factor that we included in our study was the total hospitalization charges as a surrogate to overall resource utilization. We found that there was no statistically significant difference in total hospitalization charges between groups admitted to teaching vs non- teaching facilities who received TH. Although we found higher charges in patients who are in the income group range of $43,000 to $53,999 (p=0.012), even though these groups have no difference in mortality or LOS.

Limitations

The limitation of our study is that it is a retrospective cohort study using the NIS dataset, which includes only administrative data. The authors were unable to differentiate between patients with shockable vs. non-shockable rhythms, as proper ICD-10 codes were not available for each type of cardiac arrest. There is no specific ICD 10 for out of hospital cardiac arrests. The AHA statistics showed a total of 350,000 out of hospital cardiac arrests in the year 2016. Our study using these ICD-10 codes showed about 13,000 cases. This discrepancy could be due to the nature of the database we have used in our study. Given it is an administrative database, the difference in coding could be a potential cause of capturing a lower number in this study.

Also, the NIS database has no information regarding details about factors of cardiac arrest variables such as rhythm, bystander cardiopulmonary resuscitation (CPR), location, and witnessed cardiac arrest. We were not able to adjust for these factors.

## Conclusions

Our study concluded that the teaching status of hospitals does not affect mortality or total hospitalization charges in patients with cardiac arrest who received TH. There was no effect of age, sex, race, insurance provider, or the geographical location of the hospitals on the in-hospital mortality of cardiac arrest patients who received TH. However, the utilization of TH was significantly increased in teaching hospitals. In addition, increased length of stay was found in patients admitted to teaching hospitals. Hospitalization charges were higher in the lower-income group. Further investigations are needed to explore this area of research.

## Appendices

**Table 5:** ICD codes used to identify the study population and procedure ICD-10-CM: International Classification of Disease, 10th Revision, Clinical Modification

Annexure 1	
ICD codes used to identify the study population and procedure	
ICD10-CM codes used to identify cardiac arrest patients	I46.2, I46.8, I46.9
ICD10-CM procedure codes used for therapeutic hypothermia	6A4Z1ZZ, 6A4Z0ZZ

## References

[1] Benjamin E, Muntner P, Alonso A (2019). Heart disease and stroke statistics—2019 update: a report from the American Heart Association. Circulation.

[2] Bernard SA, Gray TW, Buist MD, Jones BM, Silvester W, Gutteridge G, Smith K (2002). Treatment of comatose survivors of out-of-hospital cardiac arrest with induced hypothermia. N Engl J Med.

[3] Holzer M, Martens P, Ronie R (2002). Mild therapeutic hypothermia to improve the neurologic outcome after cardiac arrest. Hypothermia after Cardiac Arrest Study Group. N Engl J Med.

[4] Shankaran S1, Laptook AR, Ehrenkranz RA (2005). Whole-body hypothermia for neonates with hypoxicischemic encephalopathy. N Engl J Med.

[5] Hippocrates Hippocrates (1923). De Vetere Medicina. 460-375 BC. https://www.hup.harvard.edu/catalog.php?isbn=9780674991620.

[6] Nozari A, Safar P, Stezoski SW (2004). Mild hypothermic cardiopulmonary resuscitation improves outcome after prolonged cardiac arrest in dogs. Crit Care Med.

[7] Hicks SD, DeFranco DB, Callaway CW (2000). Hypothermia during reperfusion after asphyxial cardiac arrest improves functional recovery and selectively alters stress-induced protein expression. J Cereb Blood Flow Metab.

[8] D'Cruz BJ, Fertig KC, Filiano AJ, Hicks SD, DeFranco DB, Callaway CW (2002). Hypothermic reperfusion after cardiac arrest augments brain-derived neurotrophic factor activation. J Cereb Blood Flow Metab.

[9] Benson DW, Williams GR Jr, Spencer FC, Yates AJ (1959). The use of hypothermia after cardiac arrest. Anesth Analg.

[10] Polderman KH (2009). Mechanisms of action, physiological effects, and complications of hypothermia. Crit Care Med.

[11] Erecinska M, Thoresen M, Silver IA (2003). Effects of hypothermia on energy metabolism in mammalian central nervous system. J Cereb Blood Flow Metab.

[12] Zhao QJ, Zhang XG, Wang LX (2011). Mild hypothermia therapy reduces blood glucose and lactate and improves neurologic outcomes in patients with severe traumatic brain injury. J Crit Care.

[13] Nolan JP, Morley PT, Vanden Hoek TL (2003). Therapeutic hypothermia after cardiac arrest. An advisory statement by the Advanced Life Support Task Force of the International Liaison Committee on Resuscitation. Circulation.

[14] Brooks SC, Morrison LJ (2008). Implementation of therapeutic hypothermia guidelines for post-cardiac arrest syndrome at a glacial pace: seeking guidance from the knowledge translation literature. Resuscitation.

[15] Papanikolaou PN, Christidi GD, Ioannidis JPA (2006). Patient outcomes with teaching versus non-teaching healthcare: a systematic review. PLoS Med.

[16] (2018). Introduction to the HCUP National Inpatient Sample (NIS) 2016. https://www.hcup-us.ahrq.gov/db/nation/nis/NIS_Introduction_2016.jsp.

[17] Dresden SM, O'Connor LM, Pearce CG, Courtney DM, Powell ES (2015). National trends in the use of postcardiac arrest therapeutic hypothermia and hospital factors influencing its use. Ther Hypothermia Temp Manag.

[18] Abella BS, Rhee JW, Huang K-N, Vanden Hoek TL, Becker LB (2009). Induced hypothermia is underused after resuscitation from cardiac arrest: a current practice survey. Resuscitation.

[19] Merchant RM, Soar J, Skrifvars MB (2006). Therapeutic hypothermia utilization among physicians after resuscitation from cardiac arrest. Crit Care Med.

[20] Patel PV, John S, Garg RK, Temes RE, Bleck TP, Prabhakaran S (2011). Therapeutic hypothermia after cardiac arrest is underutilized in the United States. Ther Hypothermia Temp Manag.

[21] Arrich J, Holzer M, Havel C, Müllner M, Herkner H (2016). Hypothermia for neuroprotection in adults after cardiopulmonary resuscitation. Cochrane Database Syst Rev.

[22] Holzer M, Behringer W (2008). Therapeutic hypothermia after cardiac arrest and myocardial infarction. Best Pract Res Clin Anaesthesiol.

[23] Jena AB, Romley JA, Newton-Cheh C, Noseworthy P (2012). Therapeutic hypothermia for cardiac arrest: real-world utilization trends and hospital mortality. J Hosp Med.

